# Schistosomiasis at the Crossroad to Elimination: Review of Eclipsed Research with Emphasis on the Post-Transmission Agenda

**DOI:** 10.3390/tropicalmed7040055

**Published:** 2022-03-31

**Authors:** Michal Giboda, Robert Bergquist, Jürg Utzinger

**Affiliations:** 1 Institute of Parasitology, Czech Academy of Science, CZ 370 01 České Budějovice, Czech Republic; 2 Ingerod 407, 545 30 Brastad, Sweden; robert.bergquist@outlook.com; 3 Swiss Tropical and Public Health Institute, CH-4123 Allschwil, Switzerland; juerg.utzinger@swisstph.ch; 4 University of Basel, CH-4003 Basel, Switzerland

**Keywords:** acute schistosomiasis, chronic schistosomiasis, egg-induced pathology, elimination, persisting disease, post-transmission schistosomiasis

## Abstract

While chronic schistosomiasis is pathologically well defined, the acute form of the disease is less well understood. It is generally agreed that early lesions, such as lung nodules and bladder polyps, are reversible, which impedes identification of the time elapsed since exposure. The intermediate stage between the acute and the chronic forms of schistosomiasis requires further investigation, as does the clinical stage due to lesions remaining after treatment. With current schistosomiasis control efforts gradually progressing to elimination, there is a need to focus on post-transmission schistosomiasis, which not only refers to remaining lesions from previous infections, but also accounts for the potential presence of surviving worms after treatment. This issue is particularly salient for migrants from endemic to non-endemic countries and should be kept in mind for returning expatriates from schistosomiasis-endemic countries. Negative stool examination or urine filtration are generally taken as indicative of cure since rectoscopy for *Schistosoma mansoni* infection, or cystoscopy for *S. haematobium* infection, are rarely performed. However, pathology of affected organs may persist indefinitely, while potentially remaining live worms could produce additional pathology. Hence, post-transmission schistosomiasis can prevail for years after elimination of the disease, and thus, warrant further attention.

## 1. Acute and Chronic Schistosomiasis in Historical Context

Last year marked the 170th anniversary of Theodor Bilharz’s first observations of the worm in a human body in Egypt [1]. However, the question of how the infection occurred remained open until Robert Leiper, more than 60 years later, not only demonstrated the parasite’s life-cycle, but also confirmed Bilharz’s original idea of at least two different species [2]. However, the hard-won knowledge that the disease is caused by parasitic worms of the genus *Schistosoma* did not immediately help the patients since it took another 60 years before a safe and efficacious drug (i.e., praziquantel) was discovered and approved after a series of clinical trials in different settings [3,4]. Yet, loose ends still remain to date, among them a clear clinical definition of the different stages of the disease, particularly post-transmission schistosomiasis. This stage has a dual form: on the one hand, serious lesions after long-term infections remain a lifelong burden, while on the other hand, we have to consider the risk of surviving worms, sometimes for decades. A discussion of this and other open questions require a brief recapitulation of *Schistosoma* infection and the clinical stages of the disease.

Acute schistosomiasis [5] reflects the activation of the human immune system when schistosome cercariae penetrate the unbroken skin of humans during water contact and transform into schistosomula that enter the blood circulation via dermal lymphatic vessels. The parasites eventually migrate to the hepatic portal system as paired still immature male and female worms, and from there move towards their final destination in the venules around the small intestine (intestinal type) or those around the bladder (urogenital type), the former mainly caused by *S. mansoni* and *S. japonicum* and the latter by *S. haematobium*. This is where the mature schistosome worm pairs finally attach, and where the females start producing and excreting eggs destined for penetration into the intestines or the bladder to eventually reach the intermediate host that are freshwater snails. However, many eggs fail to achieve penetration and end up in various organs, mainly the liver or bladder wall. Four to nine weeks after infection marks the start of egg deposition in the host, which stimulates the immune system to respond to the foreign proteins leaking from the mature eggs in the human tissues. This reaction is followed by an immune process aimed at creating a balance between resistance to new infections on the one hand and a down-regulation of anti-egg responses on the other hand. Schistosomiasis refers both to the infection and the disease, where the latter is entirely due to immune responses against retained parasite eggs. Indeed, if all eggs were always excreted from the body, symptoms would never appear, as the adult worms avoid the immune system by covering their surface with host proteins [6].

Large intestinal worm burdens can cause anaemia due to bleeding when the eggs penetrate the intestinal wall [7,8]. Although this could also follow from digestion of host erythrocytes by the parasite [9], a more important outcome of the latter fact is that active infection can be diagnosed by demonstration of circulating, regurgitated schistosome enzymes, or indirectly through the determination of antibodies against these compounds [10]. The disease develops via an acute stage that normally lasts 5–6 weeks after the first infection but can be prolonged up to 3 months (depending on the infecting species) [11]. The acute stage refers to the situation in which non-immune individuals (e.g., very young children and tourists from non-endemic areas) are first exposed [12], but this term is sometimes also applied to re-infected individuals, particularly in the People’s Republic of China [11,13]. The severity of clinical symptoms varies according to the number of infecting parasites and the degree and type of immune responses initiated. Dry cough and slight to strong angioedema together with increased counts of eosinophil leucocytes in the blood are tell-tale signs of acute infection [11,14].

In untreated individuals, the infection changes into a lifelong stage, referred to as chronic schistosomiasis. There is still no vaccine against schistosomiasis, but treatment with the antischistosomal drug praziquantel is safe and efficacious [4,15,16]. Chemotherapy is the only remedy, but it must be given repeatedly in schistosome-endemic areas due to rapid reinfection in the absence of other control measures, such as snail control; information, education, and communication (IEC); and water, sanitation, and hygiene (WASH) [17]. Chronic schistosomiasis is the main form of the infection and reflects the outcome of immune responses against compounds escaped from the egg. There is also a stage between the two forms that has not been fully illuminated. For example, eggs may be found in stool or urine samples, while acute symptoms are also present [18]. As pointed out by Jauréguiberry and colleagues [19], this may occur when schistosomula repeatedly pass the pulmonary and hepatic portal system before finding their final destination.

Gobbi et al. [20] published an interesting idea of how to identify acute schistosomiasis regardless of the time elapsed since exposure, which is a challenge since lung nodules and/or bladder polyps appear early but do not remain long. We agree with their argument that criteria from biological, pathological, and clinical viewpoints are still not precisely defined, but argue that both the intermediate stage between the acute and the fully developed chronic schistosomiasis needs further investigation, and so does the clinical observation of new lesions appear after treatment.

Many reports note that old and even new lesions can be found in immigrants who have lived in non-endemic countries for a long time without symptoms [21,22,23,24,25,26]. This problem is not new, but it has become more common and widespread because of increased displacement of people, many of whom are refugees from countries endemic for schistosomiasis fleeing to Europe. The life schistosome span in the human host is frequently given as 3-10 years; however, examples of worm survival for 20 years or even more than 30 years have been reported [27,28].

The purpose of this review-type paper is to bring attention to the issue of post-transmission schistosomiasis [29,30], which does not only consist of lesions remaining after treatment, but can also be caused by the minority of live worms that resist praziquantel, either due to immaturity of the invading parasites at the time of treatment or nascent parasite tolerance.

## 2. Clinical Investigations

The record of publications reporting post-transmission schistosomiasis stretches back to the 1950s [27,28]. However, the number of papers reporting on this subject is increasing, particularly in the last decade [21,22,23,25,26]. For example, two papers discuss the complications of imported schistosomiasis in Spain [22,26], while two other communications refer to the same kind of problem in Italy and Israel [23,25]. Giboda [24] studied foreign students and workers, aged 18–35 years, who came between 1983 and 1989 to what was then Czechoslovakia. Together, these papers represent a 70-year record of post-transmission schistosomiasis, and they all emphasise the need for sensitive diagnostic approaches.

The first Spanish paper [22] presented a relatively small sample of sub-Saharan African immigrants with long-term residence in the European Union. Up to 90% of them presented with haematuria, dysuria, and abdominal pain, suggesting urogenital schistosomiasis. Serology was positive in 80% of the cases, while parasite eggs were only found in 10% of them, either indicating that the infection was not active or that the microscopic diagnosis was not sufficiently sensitive. A large part of these infections must have been old, since as many as 36% of the patients had chronic lesions, most of them so serious that they resulted in partial renal failure. The second Spanish paper [26] also reported observational data from sub-Saharan migrants, but this was a considerably larger study. After screening more than 3000 people, 830 patients (27%) showed positive serology for schistosomiasis, with 326 (10.5%) confirmed as infected after the detection of eggs in faeces, urine, or biopsy samples.

A study from Israel [25] discussed immigrants from East Africa focusing on hospitalised patients with a pathological or microscopic confirmation of chronic schistosomiasis. It was a small study including 32 suspected and 11 confirmed cases. Most of them (82%) presented with gastrointestinal symptoms suggestive of schistosomiasis mansoni. Sensitivity of stool smear, serology, and tissue diagnosis by histopathology or microscopy were 14%, 100%, and 89%, respectively. Standard diagnostic techniques such as microscopy for parasite eggs and parasites were not felt to be helpful for assessing chronic lesions, as they necessitated advanced procedures such as colonoscopy and/or liver biopsy. However, a diagnostic algorithm for chronic schistosomiasis was proposed to avoid unnecessary invasive procedures.

A study carried out in Italy [23] assessed differences in demographic characteristics, clinical presentation, and laboratory data including ultrasound findings in a referral centre for tropical diseases. Overall, 272 patients had acquired schistosomiasis in Africa. Symptoms were reported by 53% of the patients, with abdominal pain (36%), macroscopic haematuria (11%), and genito-urinary symptoms (7%) being the most frequent. Increased IgE and blood eosinophilia were observed in 169 (64%) and 130 (48%) of the patients, respectively. The proportion of positive serology was 250/272 (92%), while 103/272 (38%) were positive microscopically either for *S. haematobium* (48%), *S. mansoni* (47%), or both (6%). The infection was further classified based on organ involvement as follows: intestinal (18%), hepatosplenic (5%), urogenital (49%), and indeterminate (44%). Multivariate analyses showed that younger age, abnormal ultrasound findings, and blood eosinophilia were significantly associated with positive microscopy. Since many patients had positive serology but negative microscopy, it was felt that a combination of diagnostic tools, including testing for circulating schistosome antigens, should regularly be applied.

Giboda’s paper [24] referred to investigations carried out in the 1980s. We feel that it deserves to be referred in somewhat more detail, as it is a large study and describes a situation, which is becoming increasingly common in Europe. Indeed, this paper reports on 5510 individuals from a large variety of endemic countries entering Czechoslovakia between 1983 and 1989. As required at that time, they were all subjected to laboratory examination of stool, urine, blood, and, sometimes, other specific biological material, with those showing positive test results examined clinically. The infections detected were presumably chronic since the individuals had been in a non-endemic country for some time. Urogenital schistosomiasis was diagnosed by urine filtration, with the result given as the number of eggs found per 10 mL of urine [31], while the intestinal form was investigated by stool examination using the Kato–Katz thick smear method [32], and the result was reported as the number of eggs per gram of stool (EPG). The intensity of infection was categorised as light, moderate, or heavy according to guidelines put forth by the World Health Organization (WHO) [33]. Overall, 16 students from Angola were found positive for either *S. mansoni* or *S. haematobium*, while 26 students from Yemen were found to be infected with *S. mansoni* only. Seventeen of the *S. mansoni* infections discovered (54.8%) qualified as light (1–99 EPG) and 14 (45.2%) as moderate (100–399 EPG). Twelve (92.3%) of the thirteen *S. haematobium* infections were light (<50 eggs/10 mL of urine), but one student had a heavy infection (208 eggs/10 mL of urine). In contrast to urine filtration, it is known that the Kato–Katz thick smear method is not effective in individuals with light parasite loads, since its sensitivity is only satisfactorily in individuals with >100 EPG [34]. Consequently, the real prevalence of *S. mansoni* might have been considerably underestimated at that time, as it probably still is in many parts of the world [35].

In the study by Giboda [24], stool or urine samples, as well as biopsy excisions from 13 patients originally infected with either *S. mansoni*, *S. haematobium*, or both species concurrently, were examined 12–26 months after praziquantel treatment (Table 1). While parasite eggs were detected in nine patients one year or more after treatment, importantly, undamaged miracidia were detected in eggs found in some of the biopsies, thus demonstrating the presence of living worms in these patients. Reminiscent results were reported by Mosimann et al. [36] with reference to the large number of autopsies they carried out in Egypt in the 1970s. Most patients in the study by Giboda were asymptomatic and presented only light infections, which may be due to originally higher worm burdens that left a few worms alive after praziquantel treatment, as the drug is not 100% efficacious depending on various factors (e.g., dosage, species, age, and intensity of infection; as reviewed for both intestinal and urogenital schistosomiasis by Danso-Appiah et al. [37,38].

When colonoscopy was performed on 16 intestinal schistosomiasis patients before and 6 months (or later) after praziquantel treatment, patchy lesions were observed in the whole stretch of the colon, most frequently in the recto-sigmoid segment in all patients, and they only disappeared in three of them after treatment (Table 2). Although eggs were not detectable in the biopsies after therapy in about half of the patients, the granulomatous reactions were largely not reversible within the time period.

In addition, cystoscopy and ultrasound examination of the bladder were performed in nine patients with urogenital schistosomiasis. At the time of treatment, only patients with dysuria and macroscopic haematuria had polypoid lesions and focal sandy patches. After therapy, the polypoid lesions were not observed, while the sandy patches were less pronounced and had disappeared in two of the three cases after 20 months (Table 3). These clinical improvements were most probably due to the reduction of the heavy, local tissue egg burden. However, here, the thickened bladder wall persisted even 20 months after treatment. Classification of the bladder lesions was mainly recorded as stage 1. Such moderate lesions have been shown to persist in a few individuals even 4 years after treatment [39].

**Figure 1:**
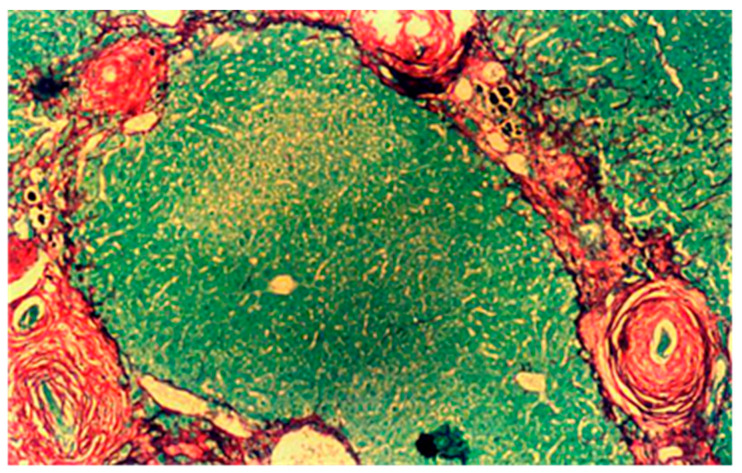
Liver section (×400) with *S. mansoni* eggs surrounded by perioval granuloma. Liver parenchyma and parasite eggs stained green surrounded by collagen I and III fibres stained red by Picro Sirius Red.

**Figure 2:**
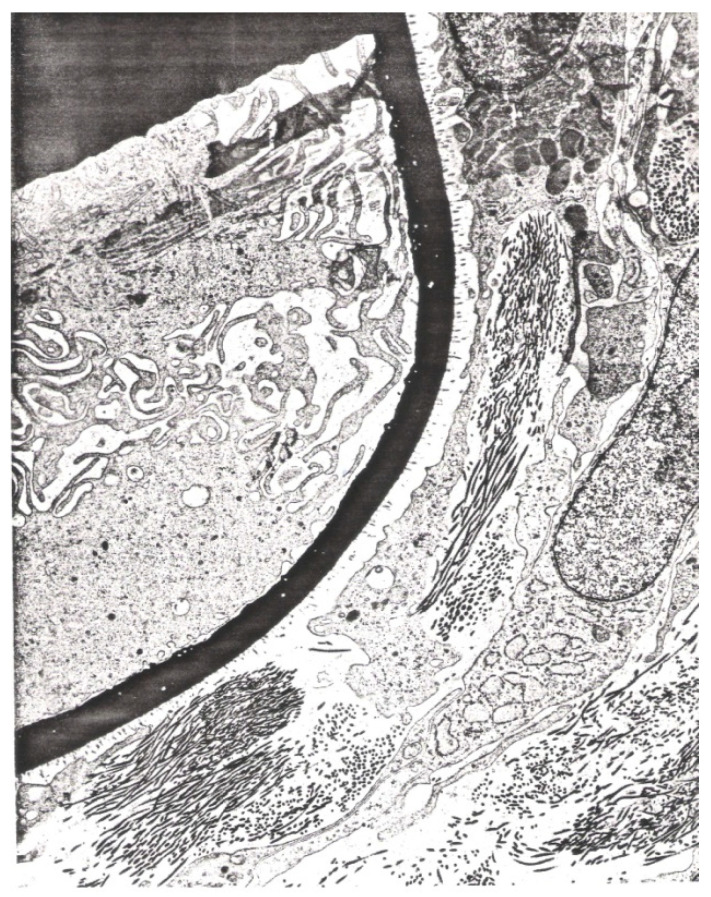
Transmission electron graph (×80,000) of the eggshell and collagen fibres. The eggshell is surrounded by many collagen fibres with part of an eosinophile leukocyte with numerous granules in view, top right.

Giboda further performed colonoscopy for *S. mansoni* infection, and cystoscopy and ultrasonography for *S. haematobium* before and after treatment with praziquantel that was administered orally under supervision with two doses 6 h apart, 40 mg/kg in total [24]. Three independent samples of stool (for *S. mansoni*) or urine (for *S. haematobium*) were examined for each patient 12 and 26 months after treatment. Biopsies for histological examinations were always taken from the site with the most marked pathological changes, either from the tunica mucosa via rectoscopy, or from tunica mucosa and tela submucosa of the bladder during cystoscopy. Paraffin sections were made and stained for microscopic visualisation of collagen I and III fibres with Picro Sirius Red [40]. Figure 1 shows a typical biopsy under the microscope. Figure 2 is added here to show additional ultrastructural information: immediately outside the thick black line in the figure (i.e., the eggshell), large numbers of collagen fibres have collected in the form of short, thin threads.

Even if egg viability in biopsy materials is important for the assessment of treatment efficacy, other techniques are also useful. Scraping of the rectal mucosa was found to be a valuable diagnostic method, as higher statistically significant positive results than that achieved by stool examination were detected in *S. mansoni* infections and the outcomes of rectal scraping were comparable to those obtained by snip biopsy from the rectum [24]. However, the former is easier, does not interrupt the continuity of mucosa, and requires no special equipment.

## 3. Research on the Schistosome Egg

What is the earliest time *Schistosoma* worms can initiate oviposition? This is not only an important question diagnostically, but has bearing on the development of the early stages of pathology. In a paper on in vitro cultivation of *S. mansoni*, Clegg [41] measured the timing of oviposition in white mice, reporting that the first eggs were produced between 34 and 35 days after infection, but he mentions that some schistosome pairs did not begin excreting eggs until 20 days later [33]. Giboda and Smith [42] conducted similar experimental infections of female C57/BL/6 mice and observed the onset of *S. mansoni* egg excretion to occur between 45 and 57 days post-infection, a seemingly different result. However, it does not necessarily contrast with Clegg’s findings, since he indicated that the worms mature within a considerable time difference and the emergence of the first eggs for individual worm pairs can indeed vary between 35 and 55 days [41].

In the study by Giboda and Smith [42], all immature eggs found matured within 9 days, at which time a first dose of praziquantel was applied, which killed 94.2% of all eggs observed, 83.2% in the small intestine and 11.0% in the liver, with a rapid drop in the egg excretion rate resulting in absence of faecal eggs and no viable eggs in the liver and intestines following a second dose of praziquantel 9 days later [42]. The data suggest that a second dose of praziquantel spaced by a few days is crucial to stop oviposition and halt pathogenesis.

**Figure 3:**
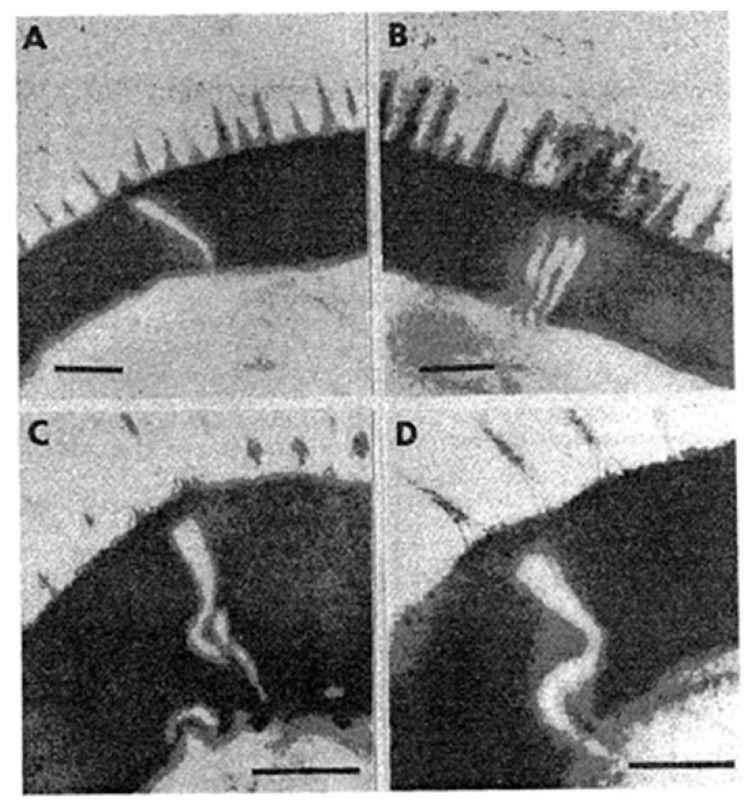
Hatching lines in the *S. mansoni* egg. (**A**) Transverse lamellae; (**B**) oblique longitudinal lamellae; (**C**,**D**) oblique transverse sections of the electron-lucid lamella in the eggshell proper. Bar length = 0.2 µm (reproduced from *Helminthologia* with permission).

The eggs of *Schistosoma* are first immature, immunologically intact, and resistant to treatment with praziquantel since the eggshell at this stage is compact and without pores conducive to antigen excretion that can induce inflammation [42]. However, as the egg matures, soluble egg antigens (SEA) secreted from its cephalic glands can pass through pores in the eggshell [43]. According to Neill et al. [44], the cytoplasmic layer (von Lichtenberg’s envelope) that is interposed between the host extracellular fluid and the developing miracidium functions as a barrier against passive diffusion. This infers that complex macromolecule, such as SEA, must undertake an active, perhaps selective, transport out of the egg. Ždárská et al. [45] studied the ultrastructure of *S. mansoni* eggs collected from the tissues of mice 67 days after infection and observed vertical, oblique, and horizontal lamellae on electron micrographs of a schistosome egg (Figure 3). Rather than forming channels to the egg surface through which SEA could be excreted, these lamellae are apparently identical to the future lines of rupture ensuing hatching of the miracidium.

**Figure 4:**
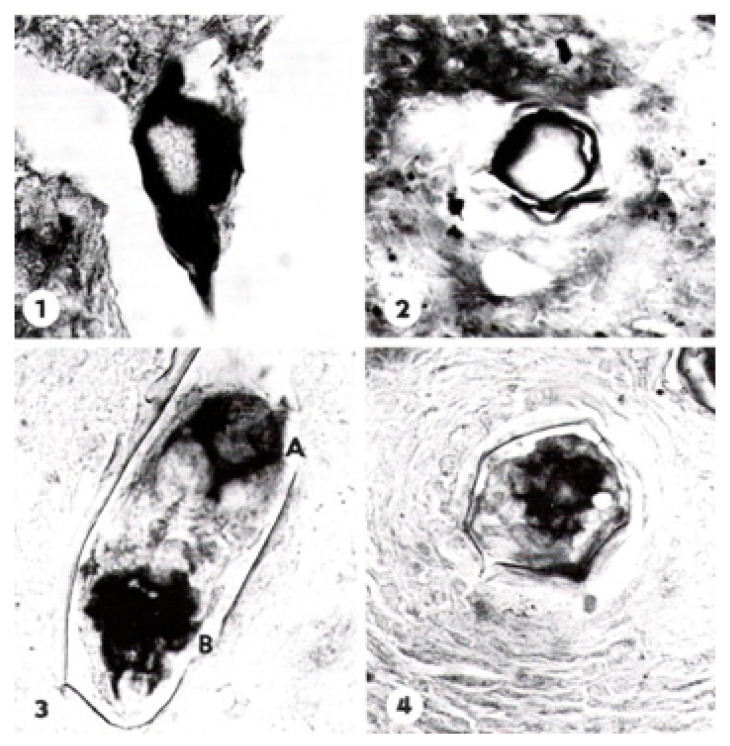
Activity of alkaline phosphatase (AP) in the body surface layer of the *S. mansoni* egg and in von Lichtenberg’s envelope stained by naphthyl AS-D phosphatase + Fast BB. (**1**) Immature eggs with developing miracidium—diagonal section (×640); (**2**) eggs with developing miracidium—transversal section (×miracidium. (500)); (**3**) The site of alkaline phosphatase changed during the course of maturation of the miracidium. AlP was demonstrated in germ cells and in the sensory endings of neural cells, located only at the anterior part of miracidium (A and B) (×780); and (**4**) eggs with fully developed miracidium—transversal section (×780) showing AP bound to the germ cells in the posterior part of miracidium (reproduced from *Folia Parasitologica (Praha)* with permission).

Giboda and Ždárská [46] developed a viability assay based on the activity of alkaline phosphatase (AP) contents in the schistosome egg, which appears early and differentiates as the egg develops. In the initial stage of maturation, the miracidium stains positive for this enzyme only on the surface and in the envelope, following the terminology of Neill et al. [44]. As seen in Figure 4, the site of AP activity changes during the course of maturation and appears first in the sensory endings of the neural cells located at the anterior, and somewhat later also in the posterior part of the miracidium. The nucleated envelope [47] in the mature egg contains an extensive endoplasmic reticulum suggestive of active protein synthesis. Furthermore, Reynolds’ layer [45] appears between the envelope and the eggshell in mature eggs only and may represent accumulated secretions [44]. The detection of AP in the egg is thus a specific and sensitive approach to differentiate between viable and dead eggs regardless of whether they are immature or mature [46]. The AP test for egg viability is considerably more sensitive compared with the more conventional hatching test, as described by Xu and Dresden [48], which only detects viable mature eggs with fully developed miracidia.

The challenge to find typical live eggs and to distinguish live eggs from others that have undergone degradation in biopsies from different tissues still confronts clinicians and pathologists. Based on schistosomiasis japonica, Gu et al. [49] investigated several different morphological and biochemical markers of *S. japonicum* to find a sensitive method that would be more practical and accurate for the clinical diagnosis of schistosomiasis. They concluded that the most sensitive and specific method was the detection of retrotransposons of *S. japonicum* genome mRNA, while they settled for the detection of succinic dehydrogenase as being simpler, more rapid, and therefore the most practical approach for clinical application.

## 4. Prospects of Elimination

The schistosomiasis burden in the world is substantial. Over 800 million people live in areas endemic for schistosomiasis, with around 230 million constantly infected [17,50,51,52]. The disease is counted as a neglected disease, but strong activities in many countries aim at eliminating the infection within the foreseeable future. Elimination is almost accomplished in northern Africa, and timely progress has been made in Brazil, Egypt, and the People’s Republic of China. However, 90% of the global schistosomiasis burden is now concentrated in sub-Saharan Africa [52]. No other species than *S. mansoni* exists in Latin America and the Caribbean islands, while *S. mansoni* and *S. haematobium* dominate in Africa. *S. japonicum* is the only species in the People’s Republic of China and the Philippines and some minor pockets in Indonesia. Cambodia and the Lao People’s Democratic Republic share a limited endemic area of *S. mekongi* [53]. *S. intercalatum* and *S. mekongi* and a few other subgroups can also parasitise humans, but *S. mansoni, S. haematobium*, and *S. japonicum* are the cause of most infections [51].

Elimination of schistosomiasis is still a distant goal for most endemic countries, though considerable progress has been made over the past decades [52,54,55]. The People’s Republic of China may be the most striking example, as it not only had to overcome the fact that *S. japonicum* is a zoonosis that infects humans and a wide spectrum of wild and domestic animals, but also by the mid-20th century was home to more than 10 million infected people [56,57]. In spite of this situation, elimination of the disease that Mao Zedong called the “God of Plague” [58] is within reach this decade [59]. Of note, Japan has actually been declared free from *S. japonicum* [60] and, apart from the People’s Republic of China, there are a few more countries that are close to eliminating schistosomiasis, such as the Islamic Republic of Iran and Morocco [61,62].

Puerto Rico is a case in point. At a latitude around 18°15′ N and longitude 66°30′ W, Puerto Rico belongs geographically to the Caribbean islands and is a United States territory with a constitution of its own. It has a rich history in research and training in tropical diseases. For instance, the School of Tropical Medicine was formally established in 1925 and existed as an independent entity until 1949, when it was integrated into the School of Medicine of the University of Puerto Rico. The occurrence of *S. mansoni* in the territory was first detected in the region of Mayagiiez in 1904 [63], but Puerto Rico has probably been endemic since the 16th century. In 1945, the overall estimated prevalence was 13.5% [64], and data from 1974 revealed a prevalence of infection of 32.7% in a rural community consisting of 1056 inhabitants in the eastern part of Puerto Rico [65]. A cross-sectional study performed in three localities from April 1995 to January 1996 within the frame of a WHO programme to eliminate schistosomiasis in the Caribbean found only 0.6% prevalence, with all infections being light [66]. Stool samples were obtained from 495 individuals, 286 of whom were aged 1–15 years (57.7%) and 219 were aged 16–75 years (42.3%). Only a 36-year-old woman and two males, aged 47 and 53 years, were found *S. mansoni* egg-positive. A year later, File et al. [67] reported *S. mansoni* infection in a Puerto Rican woman that resulted in a cervical polyp containing a pair of adult worms.

**Figure 5:**
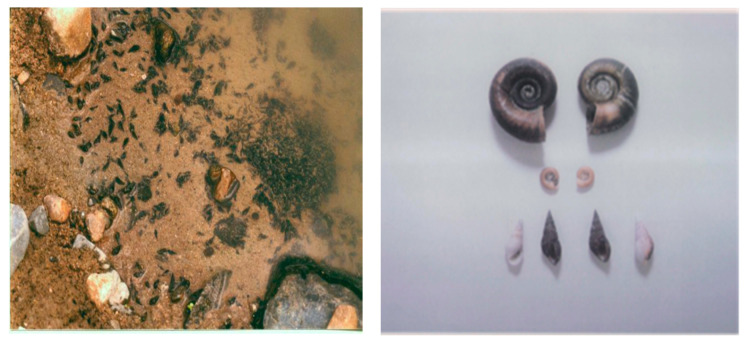
The competitive *Thiara granifera* snail in the wild (**left**) and a collection of shells (**right**), including *Marisa cornuarietis* (top), *Biomphalaria glabrata* (middle), and *Thiara granifera* (bottom).

In addition to the aforementioned stool examinations, snails were collected from May to December 1995 from 10 rivers, eight lakes, and four streams inside or in close proximity to the communities in Puerto Rico examined. Snail specimens were collected from 5-10 sites during a single visit to all freshwater bodies. This malacological survey revealed only one specimen of *Biomphalaria glabrata*, the intermediate host of *S. mansoni*, which was found dead in Canal de Guamani [66]. *Thiara granifera*, a competitive snail to *B. glabrata*, outnumbered all other snails, including the predatory snail *Marisa cornuarietis* that feeds on live or decaying plants, fish, and biological matter, including the eggs and juvenile larvae of the snail host (Figure 5). *T. granifera* feeds on algae, diatoms, and detritus and does not harm the water ecology [66].

Today, local transmission of schistosomiasis is considered interrupted as there are no longer clinical cases and no cases have been detected from surveys in recent years [68]. However, Puerto Rico has yet to be declared free of schistosomiasis, and definitive follow-up studies coupled with routine surveillance are necessary.

## 5. Post-Transmission Schistosomiasis

As we move closer to elimination of schistosomiasis in endemic countries, the disease might not disappear together with the infection, which means that we will be faced with post-transmission schistosomiasis. However, this is not a straightforward concept as it can have dual causes: either it is due to the survival of a pathology that persists after all parasites have been killed following praziquantel treatment, or a small number of schistosomes survive therapy because of inborn tolerance in some of the worms or because praziquantel is generally not 100% efficacious [37,38,69].

### 5.1. Long-Term Survival of Schistosome Worms

There is an obvious association between chronic schistosomiasis and immigrants moving from endemic to non-endemic countries. Two approaches to obtain parasitological data can be applied: (i) active case detection (ACD), which theoretically allows identification of all infected individuals regardless of clinical signs or intensity of infection, and (ii) passive case detection (PCD), which identifies only those who seek medical care due to symptoms. Regarding PCD, many cases are missed, mostly due to the absence of clinical signs symptoms and low sensitivity of diagnostic methods, particularly at low intensity of infection. The study of imported schistosomiasis to the Czech Republic referred to above [24] was based on ACD, which meant that all immigrants were compulsory examined and the parasite load quantified. Although the expression was not coined until much later, these positive cases were by definition cases of post-transmission schistosomiasis [27,28]. The experiences with more than 5500 foreigners subjected to ACD were discussed in a meeting held in October 1998 in Puerto Rico that was organised by the UNICEF/UNDP/World Bank/WHO Special Programme for Research and Training in Tropical Diseases (TDR) [30]. The proceedings of the meeting highlight the fact that schistosomiasis is not always cured by praziquantel.

Probably the first published evidence of post-transmission schistosomiasis comes from Polish refugees who immigrated to Western Australia between 1950 and 1953 after having contracted schistosomiasis mansoni in refugee camps in East Africa. Ten patients were diagnosed with live *S. mansoni* worms after an unbroken period of 20 years in Australia, and an additional five patients who had resided there for more than 30 years [27]. The diagnosis was initially suspected in five cases by various findings, such as eosinophil myelocytes in the bone marrow, radiological evidence of calcification of the bladder wall, bleeding from the ureters, cystoscopy findings of sandy patches in the bladder, discovery of ova in the wall of a fallopian tube during ectopic gestation, the presence of ova and an adult worm with leiomyoma, and a benign tumour originating in the smooth muscle cells of the myometrium [27]. Ten of these patients had eosinophil counts greater than 0.3 × 10^9^/L, three had hypersplenism, one who showed no peripheral eosinophilia had numerous eosinophil myelocytes in the bone marrow, and four were asymptomatic [27].

Additional evidence of post-transmission schistosomiasis stems from a Portuguese soldier who was infected with *S. mansoni* 34 years before returning from Angola and was harbouring an active infection since live miracidia were found in the ova excreted by faeces [28]. Another Portuguese soldier was found infected with *S. haematobium* 40 years before returning from Mozambique. He suffered from urothelial cancer but all ova were already calcified [28].

### 5.2. Post-Transmission Schistosomiasis after Elimination of the Infection Risk

The situation after elimination is quite a special one as we deal here with large numbers of successfully treated patients now who used to live in endemic areas but now reside non-endemic surroundings where there is no risk of reinfection. Since very few countries have so far been declared free of schistosomiasis, we have little evidence on which to base any post-transmission schistosomiasis approach. However, Japan provides a possibility, as this country successfully defeated their schistosomiasis problem 45 years ago. The last, new human infection in Japan was in 1977 [60], and since then, quite a few cases with remaining morbidities have been reported [70,71,72].

**Figure 6:**
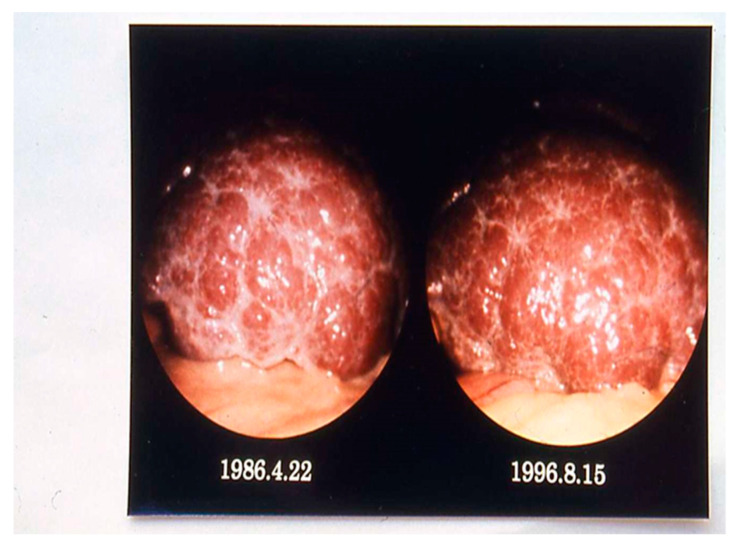
Laparoscopy of a schistosomiasis patient carried out twice with a 10-year intermission. Liver enlargement and less pronounced fibrosis with mild, irregular groove-like septa can be seen in the later investigation (Courtesy, Prof. S. Hayashi).

Although nearly 1900 patients with active or chronic liver disease have undergone diagnostic laparoscopy in the hospital in Tokyo, far from the endemic area, only nine patients with chronic schistosomiasis were found, aged 52–68 years [70]. These nine patients were analysed and compared with respect to hepatic ultrasonography, computer-assisted tomography (CT), and histology. None of them excreted any parasite eggs, and all were negative with respect to schistosome infection by immunoelectrophoresis, while the circumoval precipitin test (COPT) [73] was positive in two cases. Tests for hepatitis B virus surface antigen were negative in all cases, while antibodies against hepatitis C virus were found in four cases. In all nine patients, laparoscopy revealed yellowish, small speckles over the liver surface, later found to be subcapsular calcified S. japonicum ova. While the liver surface was almost smooth in mild schistosomiasis, multiple whitish markings and irregular, relatively wide grove-like septa were seen in more advanced cases. The liver surface of chronic schistosomiasis japonica patients appeared stable without change over more than 10.5 years after initial laparoscopy (Figure 6).

Various non-invasive diagnostic methods, such as abdominal ultrasonography and abdominal CT, have made considerable progress contributing to a deeper understanding of chronic schistosomiasis. The role of hepatic viral infection is more important than that of schistosomal infection in promoting the development of hepatocellular carcinoma.

## 6. Discussion

Smallpox is the only human infectious disease eradicated so far, leaving polio as the last century’s great scare. The development of polio vaccines by Salk [74] and Sabin [75], first used in 1955, soon wiped out this infection as well; only two countries still remain endemic (https://www.cdc.gov/polio/progress/index.htm, accessed on 10 February 2022). On the other hand, many people had remaining lesions caused by polio as late as the end of the 1900s, naturally less now as more than 65 years have passed since the first vaccination drives. This infection is a strong reminder that chronic diseases cast a shadow far into the future. Apart from schistosomiasis, there are many other chronic diseases, e.g., lymphatic filariasis, onchocerciasis, and dracunculiasis, reminding us that they cannot be written off as soon as effective treatments become available.

The detection of antibodies to different worm components, mostly the soluble egg antigen (SEA), is not only one of the most sensitive approaches we know, but the techniques used are also simple, rapid, and practical. However, the investigator is left with the knowledge that the patient in question has been in contact with schistosomes, and there is no information on when infection occurred or if the infection is still active. Antibody detection is therefore only useful as a screening technique, which must be followed up with a parasitological test or, preferably with a test of circulating antigens since egg detection is limited in sensitivity by the number of slides the microscopist can process.

Unperturbed by the historic disinterest in the diagnostic aspects of circulating antigens, Deelder and co-workers continued their line of research on the anodic (CAA) and the cathodic antigen (CCA) as markers for infection [76,77]. The antigen they worked on is found in both blood and urine and resulted eventually in the point-of-care (POC)-CCA assay [78] for the diagnosis of *S. mansoni* infection. The advantage of this test is not just its high sensitivity, but the fact that the result is not limited to the sample provided and reflects the worm load in the subject as a whole, something that will be needed for certification of eradication. Efforts to replace the Kato–Katz method for intestinal schistosomiasis, and urine filtration for the urogenital form, have been ongoing since long before the polymerase chain reaction (PCR) [79] made antigen detection sufficiently sensitive. The success of POC-CCA assay [80,81] has made WHO include in its current guidelines a recommendation to add testing for *S. mansoni* by circulating schistosome antigens [50]. WHO has an important role to play in directing additional studies to adapt the POC-CCA assay also for *S. haematobium* and *S. japonicum*, the former because mixed infections are commonplace in Africa, the latter due to the prerequisite for improved diagnostics in the People’s Republic of China, which has now entered the elimination phase.

Elimination of schistosomiasis is currently becoming achieved in a growing number of geographical regions. Although this is a vision for the immediate future, the pathology left from previously cured chronic infections would persist for many more years [70,71,72], in some areas presumably affecting large numbers of people. This is a major problem that needs to be addressed already at this point in time. Just the diagnostics required could not easily be applied widely, as this would require CT and ultrasonography in many cases. The scientific community is already aware of the quandary of female genital schistosomiasis [82] and neurological symptoms due to eggs trapped in the spinal canal [83], which represent just two of the many various types of post-transmission schistosomiasis. Another issue is the risk of leaving live schistosomes after treatment, a predicament that can only be brought down by highly sensitive diagnostics. This risk is particularly high with regard to intestinal schistosomiasis for which the Kato–Katz stool examination is still widely used in spite of missing large numbers of light infections. There is a need to rapidly widen the use of POC-CCA and DNA-based diagnostics, as the high sensitivity of these new techniques would minimise lesions due to any remaining light infections.

Importantly, post-transmission schistosomiasis is not mentioned in any current guidelines, neither by WHO nor the Centers for Disease Control and Prevention (CDC) in the United States; and not by the Chinese Center for Disease Control and Prevention (China CDC).

## 7. Conclusions

With many countries rapidly approaching the elimination of schistosomiasis, and some already having achieved it, there is a pressing need to plan for the post-transmission phase. Even if the high sensitivity of new techniques would sharply lower the risk of lesions due to any remaining live worms, the potentially large number of people with chronic disease is a major problem that will not go away. This reality must be considered without delay as we are moving ever closer to the elimination of transmission of schistosomiasis in large parts of the current endemic areas.

## Figures and Tables

**Table 1:** Distribution of *Schistosoma* eggs found among 5510 foreign students and workers screened upon entry into Czech Republic between 1983 and 1989 (adapted from Giboda [24]).

Species	In Faeces or Urine			In Biopsies		Number of Cured from All Treated
	Absence of Eggs	One Year after PZQ	Two Years after PZQ	One Year after PZQ	Two Years after PZQ	
*S. mansoni*	3	0	1	1	1	3/6
*S. haematobium*	1	0	0	0	1	1/2
Both	0	1 ^1^	0	1 ^2^	3 ^1^	0/5
Total	4	1	1	2	5	4/13

^1^*S. haematobium*; ^2^*S. mansoni*; PZQ, praziquantel.

**Table 2:** Lesions in the colon of *S. mansoni* patients before and after treatment with praziquantel (adapted from Giboda [24]).

Location of Patchy Lesions in the Colon	Patients with Lesions	
	Before PZQ	After PZQ
Ascendens	2	2
Transversum	4	2
Descendens	8	6
Rectosigmoid	16	13

PZQ, praziquantel.

**Table 3:** Bladder lesions in *S. haematobium* patients before and after treatment with praziquantel (adapted from Giboda [24]).

Months after PZQ	No. of Patients Tested	Symptom					
		Haematuria	Dysuria	Hyperaemia	Sandy Patches	Polypoid Lesions	Bladder Index ^1^
0	3	Yes	Yes	Diffuse	Focal	Focal	Stage 1
0	3	No	No	Diffuse	Diffuse	None	Stage 1
6–15	3	No	No	Focal	Focal	None	Stage 1
6–15	6	No	No	None	Focal	None	Stage 1
20–26	1	No	No	Focal	Diffuse	None	Stage 1
20–26	2	No	No	None	None	None	Stage 1

^1^ According to Laurent et al. (1990) [39]; PZQ, praziquantel.

## Data Availability

Not applicable.
